# YOLO-Citrus: a lightweight and efficient model for citrus leaf disease detection in complex agricultural environments

**DOI:** 10.3389/fpls.2025.1668036

**Published:** 2025-10-14

**Authors:** Wanmei Feng, Junyu Liu, Zhen Li, Shilei Lyu

**Affiliations:** College of Electronic Engineering (College of Artificial Intelligence), South China Agricultural University, Guangzhou, China

**Keywords:** citrus leaf disease detection, YOLOv11s, YOLO-Citrus, C3K2-STA, ADown, Wise-Inner-MPDIoU, lightweight

## Abstract

Accurate and efficient detection of citrus leaf diseases is crucial for ensuring the quality and yield of global citrus production. However, many existing agricultural disease detection methods face significant challenges, including overlapping leaf occlusion, difficulty in identifying small lesions, and interference from complex backgrounds. These limitations often lead to reduced accuracy and efficiency of object detection. Moreover, current models generally necessitate significant computational resources and possess substantial model sizes, which restrict their practical applicability and operational convenience. To tackle these issues, this study presents a novel model named YOLO-Citrus. It is a lightweight and efficient YOLOv11-based model designed to enhance the precision of detection while simultaneously minimizing computational expenses and the size of the model. This makes it more suitable for practical agricultural applications. The proposed solution incorporates three major innovations: the C3K2-STA module, the ADown module, and the Wise-Inner-MPDIoU loss function. In particular, YOLO-Citrus utilizes Star-Triplet Attention by embedding Triplet Attention into the Star Block to enhance bottleneck performance in C3K2-STA. It also adopts the ADown module as a lightweight and effective downsampling strategy and introduces the Wise-Inner-MPDIoU loss to facilitate optimized bounding box regression and enhanced detection accuracy. These advancements enable high detection accuracy with substantially reduced computational requirements. The experimental results demonstrate that YOLO-Citrus attains 96.6% mAP@0.5, representing an improvement of 1.4 percentage points over the YOLOv11s baseline (95.2%). Furthermore, it reaches 81.6% mAP@0.5:0.95, i.e., an enhancement of 1.3 percentage points compared to the baseline value of 80.3%. The optimized model delivers considerable efficiency gains, with model size reduced by 25.0% from 19.2 MB to 14.4 MB and computational cost decreased by 20.2% from 21.3 to 17.0 GFlops. Comparative analysis has confirmed that YOLO-Citrus performs better than other models in terms of comprehensive detection capability. These performance enhancements validate the model’s effectiveness in real-world orchard conditions, offering practical solutions for early disease detection, precision treatment, and yield protection in citrus cultivation.

## 1 Introduction

The citrus industry, one of the most prominent fruit sectors, performs a pivotal function within the overarching framework of the contemporary agricultural economy (Dananjayan et al., 2022). It is not only a key component of people’s daily diet but also a significant source of income for farmers. Nevertheless, current citrus cultivation is commonly threatened by various diseases, including citrus canker, Huanglongbing (HLB), rust, and melanose. These diseases cause significant yield and fruit quality reductions, which in turn lead to substantial economic losses for growers (Abdulridha et al., 2019). In recent years, it has been reported that citrus diseases result in huge global losses. With citrus canker, growers report losses exceeding 1 billion USD annually in China, while the diseases also cause yield reduction exceeding 50% in certain regions of Brazil. HLB is prevalent in Asia and the Americas and is a constant threat to lemon and sweet orange cultivations (Ali et al., 2023). Out of all major diseases, citrus canker causes loss of leaves, early fruit detachment, twig dieback, and heavy blemishing of the citrus fruit, while HLB causes plugging of the nutrient transport, root decline, canopy dieback, and huge decreases in both the yield and quality of the fruit (Cifuentes-Arenas et al., 2022). The leaves of citrus serve as the primary sites for disease occurrence. Therefore, the early detection and accurate identification of these diseases are very important for their effective prevention and control. Conventional methods used for the detection of plant leaf diseases are based on manual inspection and observation of lesions on leaves (Barbedo, 2016). As the production scale increases, these methods become time-consuming and more sensitive to various external conditions such as weather and environmental factors, which result in low accuracy and efficiency (Ferentinos, 2018). To solve these problems, intelligent detection techniques based on computer vision and deep learning are employed to enhance the precision and effectiveness of citrus disease detection (Kamilaris and PrenafetaBoldu´, 2018).

Recent advancements in computer vision and deep learning technologies have led to significant breakthroughs in leaf disease detection. These developments suggest automated identification of disease types, early-stage symptom recognition, and large-scale monitoring of plant health conditions (Wang et al., 2022). Convolutional neural network (CNN)-based object detectors are mainly classified into two types: two-stage and single-stage detectors (Luo et al., 2024). Two-stage detectors have gained significant interest owing to their superior performance in terms of precision and stability. For example, Alruwaili et al. (2022) proposed a real-time Faster Region Convolutional Neural Network (RTF-RCNN) model, which takes advantage of both static images and real-time video streams to detect leaf diseases in tomato plants. The RTF-RCNN model has obtained good performance for both detection accuracy and robustness compared to AlexNet and CNN models. Although two-stage detectors achieve high accuracy for leaf disease detection, these detectors are time-consuming during inference and resource-consuming, making them impractical for real-time applications that require fast response, such as orchards. Compared to two-stage detectors, single-stage detectors such as YOLO are more applicable to these tasks (Mo and Wei, 2024; Li et al., 2022; Xue et al., 2023; Gao et al., 2024; Zhang et al., 2022; Khan et al., 2025) because they have faster inference speed and can still achieve relatively good performance. For instance, Zhu et al. (2025) designed CBACA-YOLOv5 by integrating multiple attention and upsampling modules into YOLOv5s. Specifically, they applied the convolutional block attention module (CBAM), coordinate attention (CA), and the CARAFE upsampling module to enhance the detection of small, asymmetric, and occluded disease features in citrus leaves. The enhanced model is beneficial for feature extraction and fusion and can be applied in real-time intelligent agricultural robots. Therefore, single-stage detectors are more applicable to real-time detection applications in dynamic orchards.

Inevitably, the complexity of deep learning models gradually rises, and higher demands for computational resources and storage space emerge, which will be limited in practice. Therefore, it is necessary to optimize the lightweight design of YOLO models to improve their applications in limited resources, such as edge devices and mobile phones. For instance, Li et al. (2023) applied the GhostNet backbone and depthwise separable convolution instead of the backbone of YOLOv4, which greatly reduced the computational complexity and model parameters. The optimization model proposed in their method has a fast inference speed and low computational overhead, which is suitable for real-time deployment in tea-picking robots. Lyu et al. (2023) also optimized the YOLOSCL model for detecting citrus psyllids based on YOLOv5s. By compressing the network and lowering the parameters, the model obtains higher detection accuracy and can be mounted on the Jetson AGX Xavier edge computing platform. The lightweight design of the aforementioned models plays a crucial role in enhancing deployment efficiency and reducing computational resource demands (Han et al., 2022; Zeng et al., 2023; Cui et al., 2023). However, how to balance detection accuracy with computational efficiency while maintaining a lightweight design remains an open challenge.

Moreover, the Intersection over Union (IoU) metric used in the YOLO series is based solely on the geometric overlap of bounding boxes, which constrains its sensitivity in lesion localization (Li et al., 2024; Ji et al., 2023). This limitation is especially evident under conditions of leaf occlusion or blurred lesion boundaries, such as the diffuse margins observed in canker disease lesions. As a result, the model becomes susceptible to missed detections and localization drift. Consequently, there is an urgent need to introduce methods such as dynamic shape constraints or edge feature enhancement to improve localization accuracy and robustness in complex scenarios (Abulizi et al., 2024).

To address the aforementioned technical challenges, this study proposes YOLO-Citrus, a lightweight and improved model based on the YOLOv11s architecture. It is designed to enhance citrus disease detection in complex orchard environments characterized by uneven lighting, dense foliage occlusion, overlapping fruits, and varying background conditions. The core innovations of our proposed approach are outlined as follows:

- Data augmentation and expansion: The data enhancement tools provided by the Roboflow platform are utilized to perform processing operations, including image rotation, scaling, flipping, and brightness adjustment, on the acquired images of diseased citrus leaves. The augmented data improve the generalization and robustness of the target detection model, enabling it to be applied to various datasets.
- C3K2-STA (C3K2-Star-Triplet Attention) module: To enhance feature extraction capability and reduce computational complexity, the C3K2-STA module is designed by integrating the Star Block structure and the Triplet Attention mechanism into the C3K2 architecture. This module improves the inference performance of C3K2, reduces redundant computation, and enhances the effectiveness of feature representation.
- ADown module: The ADown module is designed as a downsampling component in the proposed model. Average pooling and max pooling are combined with the ADown module to extract global and local features. Meanwhile, background and edge information are enhanced by feature segmentation and concatenation of the ADown module. In addition to that, the ADown module can also greatly reduce the number of parameters and computational complexity and improve the inference efficiency of the model.
- Wise-Inner-MPDIoU: To improve the accuracy and stability of bounding box regression, a novel loss function named Wise-Inner-MPDIoU is introduced as a replacement for the original Complete Intersection over Union (CIoU) loss in YOLOv11. This loss function adopts the weighting strategy of Weighted IoU (WIoU) and the corner distance constraint of MPDIoU. To improve the localization of the bounding box more accurately, an inner product distance constraint is introduced. By allocating different weights in different situations, Wise-Inner-MPDIoU highlights key bounding boxes and minimizes the impact of position deviation. Meanwhile, the distance between predicted boxes and ground truth boxes is also minimized. The object localization capability of the model is greatly improved, and the convergence rate in a complex agricultural scene is greatly accelerated.

## 2 Dataset description

### 2.1 Data acquisition

**Figure 1:**
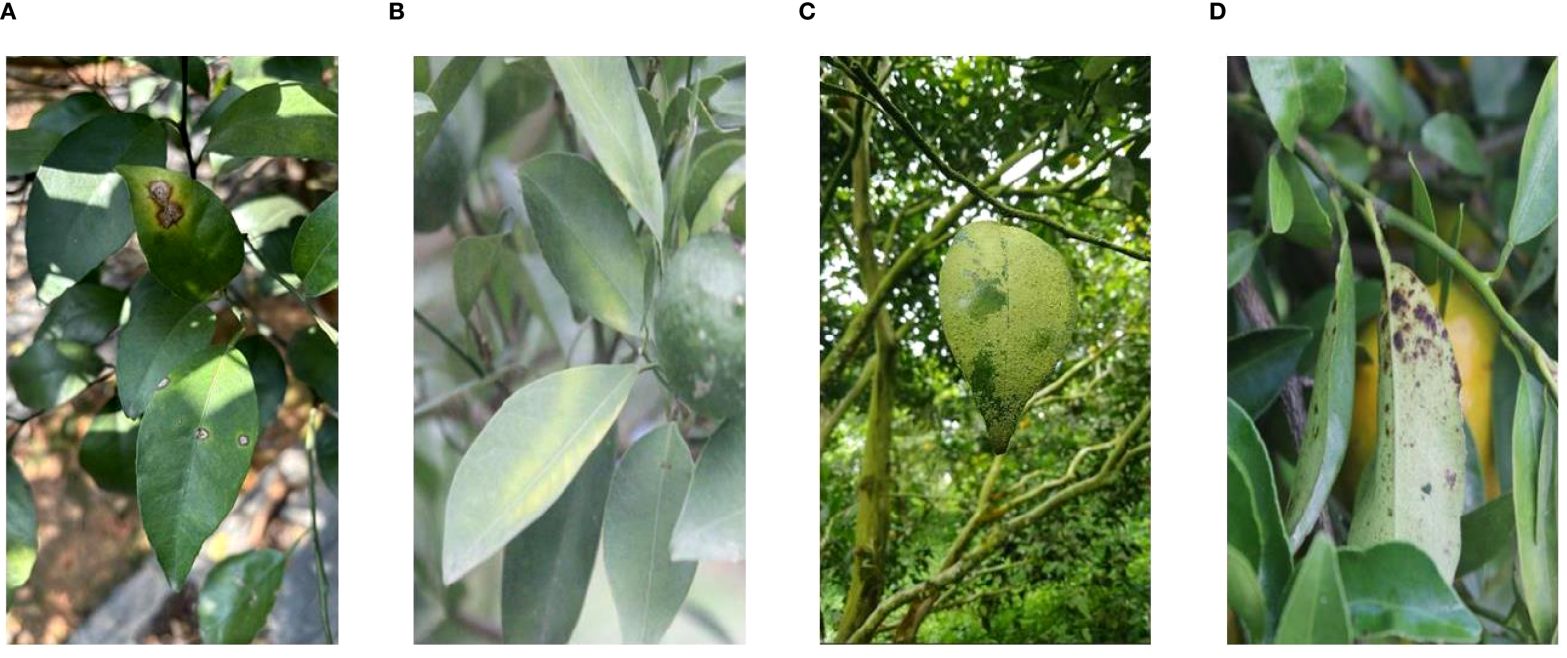
Some representative samples of our dataset. **(A)** Canker, **(B)** HLB, **(C)** rust, and **(D)** melanose. HLB, Huanglongbing.

The dataset for citrus disease detection in this study is mainly collected from the citrus orchard experimental base of South China Agricultural University (Guangzhou, Guangdong Province, China). It covers various disease types such as canker, Huanglongbing, rust, and melanose. Some representative images from the dataset are displayed in **Figure 1**. Data collection is carried out from June to December. In the process of taking images, both mirrorless interchangeable-lens cameras (Canon R8) and handheld cameras (iPhone 15 Plus) are employed as the shooting equipment. The shooting distance is controlled between 30 and 100 cm to capture the characteristics of citrus diseases. The main shooting environment is natural light on sunny days, and the shooting time is chosen between 10:00–11:30 a.m. and 2:30–4:00 p.m. In these two time periods, the lighting is relatively stable, which reduces the impact of intense illumination conditions and makes the image quality more similar for subsequent enhancement and processing tasks.

### 2.2 Data preprocessing

**Figure 2:**
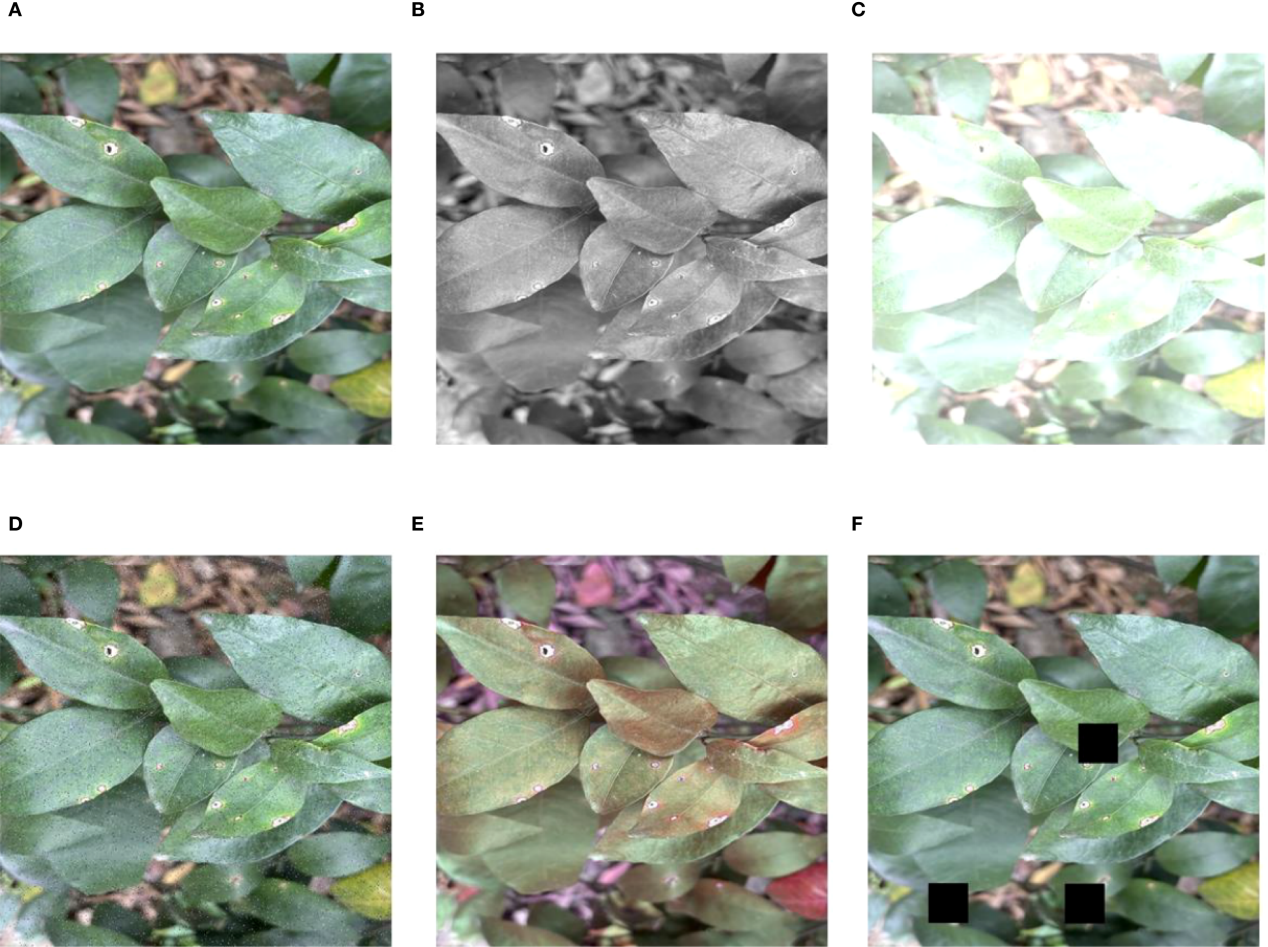
Effect of image enhancement on dataset. **(A)** Original, **(B)** grayscale, **(C)** brightness, **(D)** noise, **(E)** hue, and **(F)** cutout.

The annotation process is conducted using the LabelImg tool to label the regions affected by citrus diseases, which ensures the precise representation of both the location and category of each disease in every image. Then, the annotated data are randomly divided into training, validation, and test sets in an 8:1:1 ratio, which consist of 1,046, 131, and 131 images, respectively. In order to increase the diversity of the data and make the model more robust, data augmentation techniques are applied to the dataset. These methods convert images into grayscale to simulate different lighting conditions and adjust the brightness to simulate varying light intensities. In addition, Cutout is adopted to cover some areas of the image randomly so as to make the model more adaptable to the absence of information. Additionally, noise is added to make the model more robust to interference, and random hue augmentation is used to increase the diversity of color change. The effect of image enhancement on the dataset via the image enhancement techniques is illustrated in **Figure 2**. All images are uniformly resized to 640 × 640 pixels to meet the input specifications of the YOLO model and ensure consistency across the dataset. After applying the aforementioned data augmentation methods, the original 1,308 images are expanded to a total of 3,808 images. Specifically, the number of samples per category increased to 1,026 for canker, 882 for HLB, 714 for rust, and 711 for melanose. These augmentations greatly improve the diversity of the dataset and enhance the ability to identify diseases in various imaging conditions (Lin et al., 2025; Al-Masni et al., 2018).

## 3 Method

### 3.1 The YOLOv11 network structure

**Figure 3:**
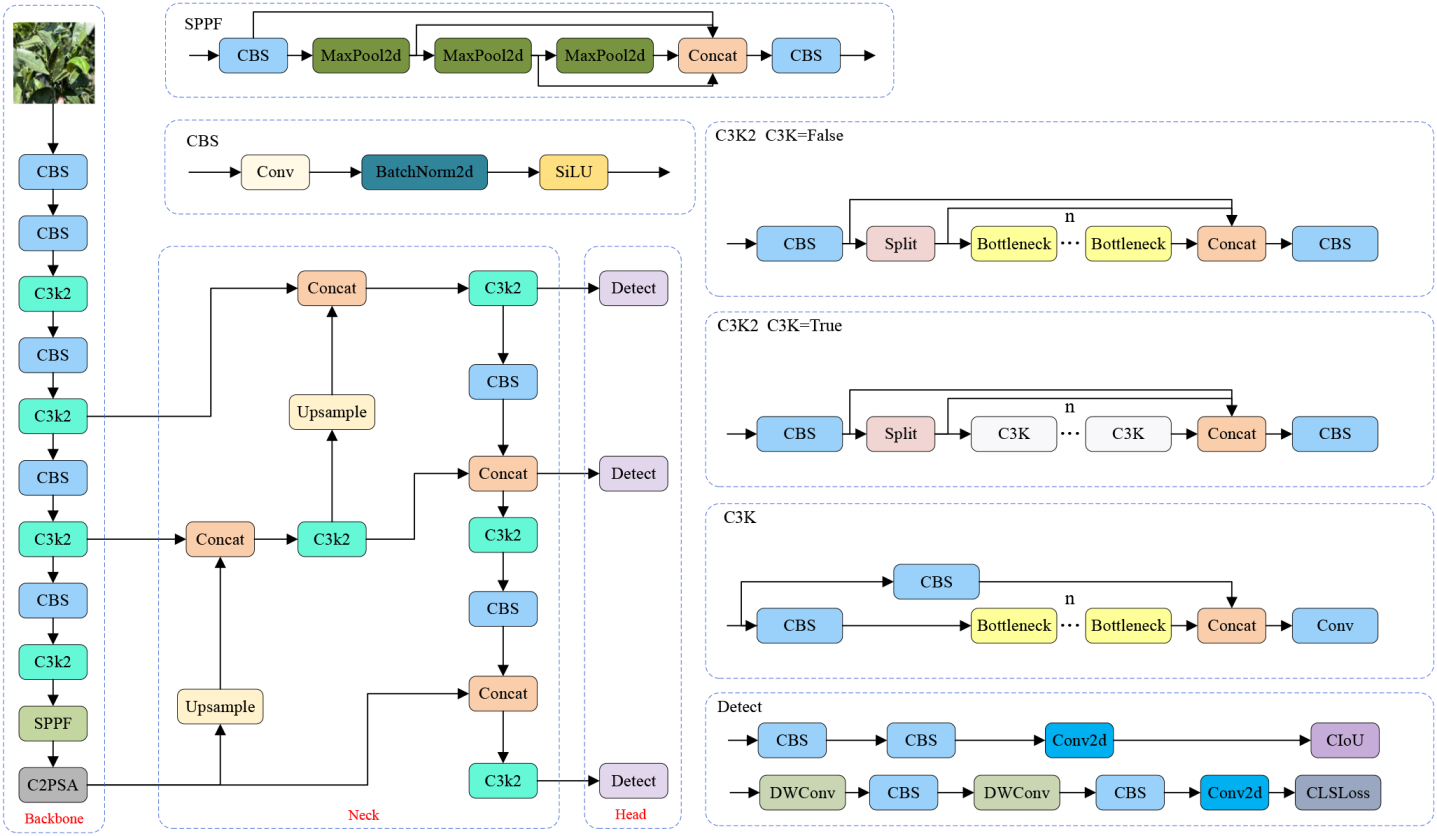
The YOLOv11 network architecture.

YOLOv11 is a newly developed and efficient object detection algorithm introduced by the Ultralytics team. This version inherits the excellent characteristics of the YOLO series algorithms. It is applicable in scenes with high requirements of precision and real-timeness (Khanam and Hussain, 2024). Compared to YOLOv8, YOLOv11 introduces several improvements. In particular, the C2f module is substituted by the C3K2 module, which enhances feature extraction by adjusting the convolutional layer configuration and incorporating a more efficient cross-stage feature interaction mechanism. Furthermore, a C2PSA module is appended after the SPPF module and connected to the backbone network of YOLOv11, which improves the ability to integrate multi-scale features. In the detection head, YOLOv11 keeps the anchor-free idea of YOLOv8 and introduces a dynamic gradient allocation module. By adjusting the loss weights of classification and regression adaptively, the contradiction between target localization and classification in involved scenes is alleviated. The structure of the whole network of YOLOv11 is shown in **Figure 3**.

### 3.2 Overview of our network

**Figure 4:**
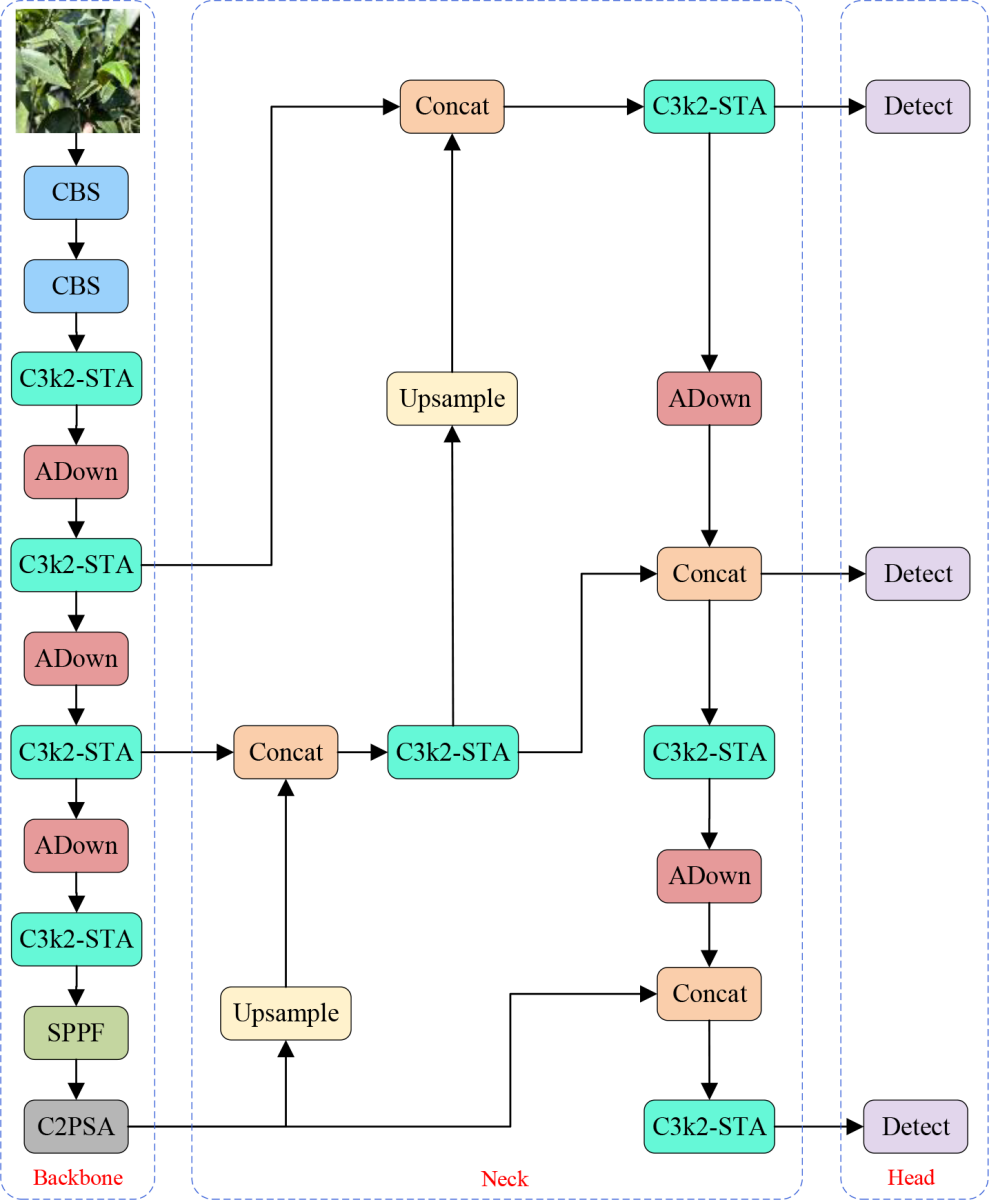
The YOLO-Citrus network architecture.

To accurately detect citrus leaf diseases in complicated agricultural scenarios with low computational costs, this paper presents an extension of the YOLOv11 algorithm, named YOLO-Citrus. By designing the C3K2-STA module, the ADown downsampling strategy (Tong et al., 2024), and the Wise-Inner-MPDIoU loss function (Tong et al., 2023; Zhang et al., 2023; Ma and Xu, 2023), YOLO-Citrus overcomes the multiple challenges existing in agricultural scenarios, such as leaf occlusion, tiny disease spot detection, and complicated background disturbance. In particular, as for the C3K2-STA module, it integrates dynamic receptive field modulation with a cross-dimensional attention mechanism to strengthen the discrimination of leaf texture features and disease edge characteristics. The ADown module utilizes the two-mode pooling strategy and axial feature reorganization to preserve delicate disease information and reduce computational cost. Moreover, the Wise-inner-MPDIoU loss function enhances the localization accuracy of irregularly shaped leaf lesions by introducing geometric constraints and a dynamic weight strategy. For orchards with overlapping leaves, non-uniform brightness, and environmental noise scenarios, YOLO-Citrus can extract effective leaf features in real-time and conduct disease pattern analysis. The global structure of YOLO-Citrus is displayed in **Figure 4**.

### 3.3 C3K2-STA

**Figure 5:**
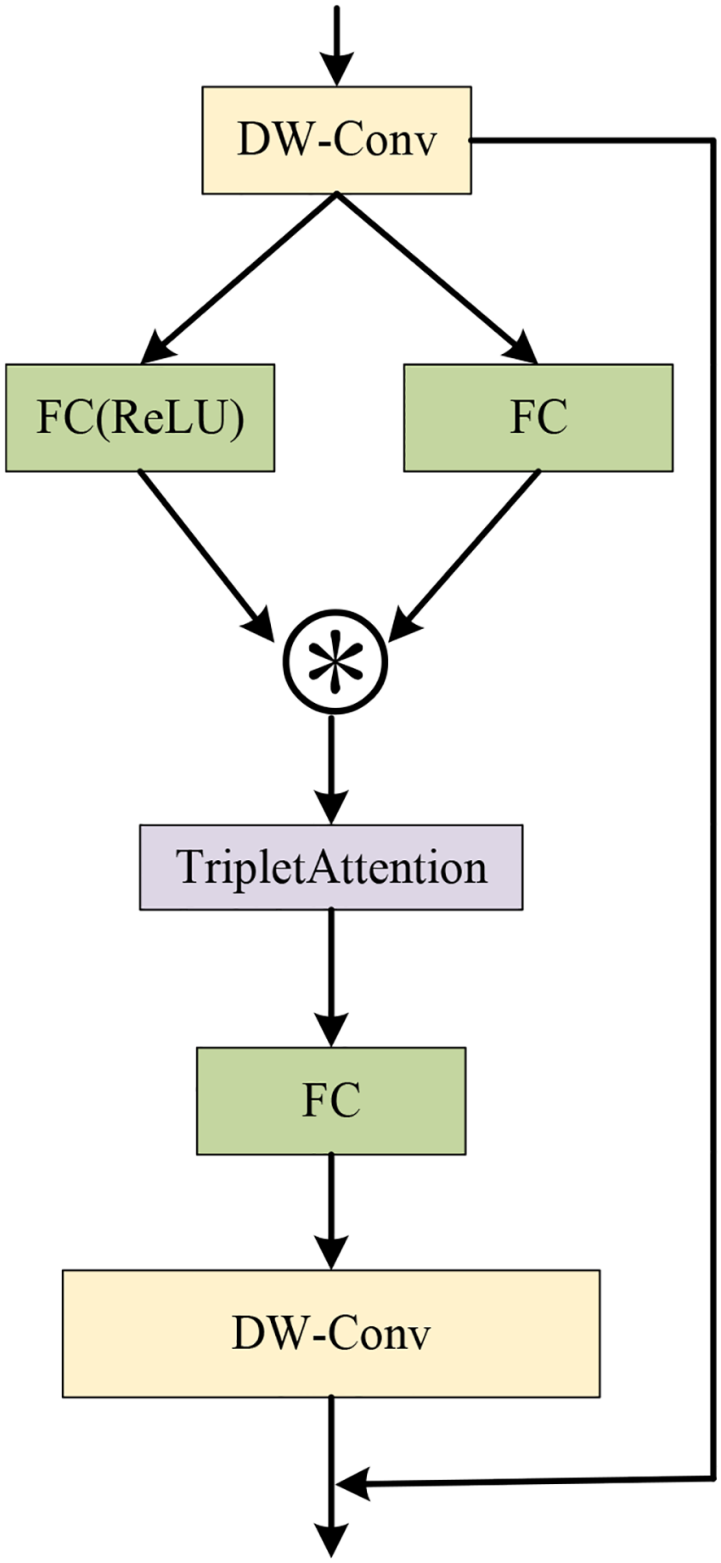
The Star-Triplet Attention block.

To address the limitation of neural networks in feature extraction while keeping the network lightweight, the C3k2-STA module is proposed. Not only can the module improve the inference performance of the model, but it could also decrease the number of model parameters and computation greatly. Specifically, the Triplet Attention mechanism (Misra et al., 2021) is incorporated into the Star Block of the StarNet (Ma et al., 2024) framework to form the Star-Triplet Attention, as illustrated in **Figure 5**. Finally, this designed block replaces the original BottleNeck module in C3.

**Figure 6:**
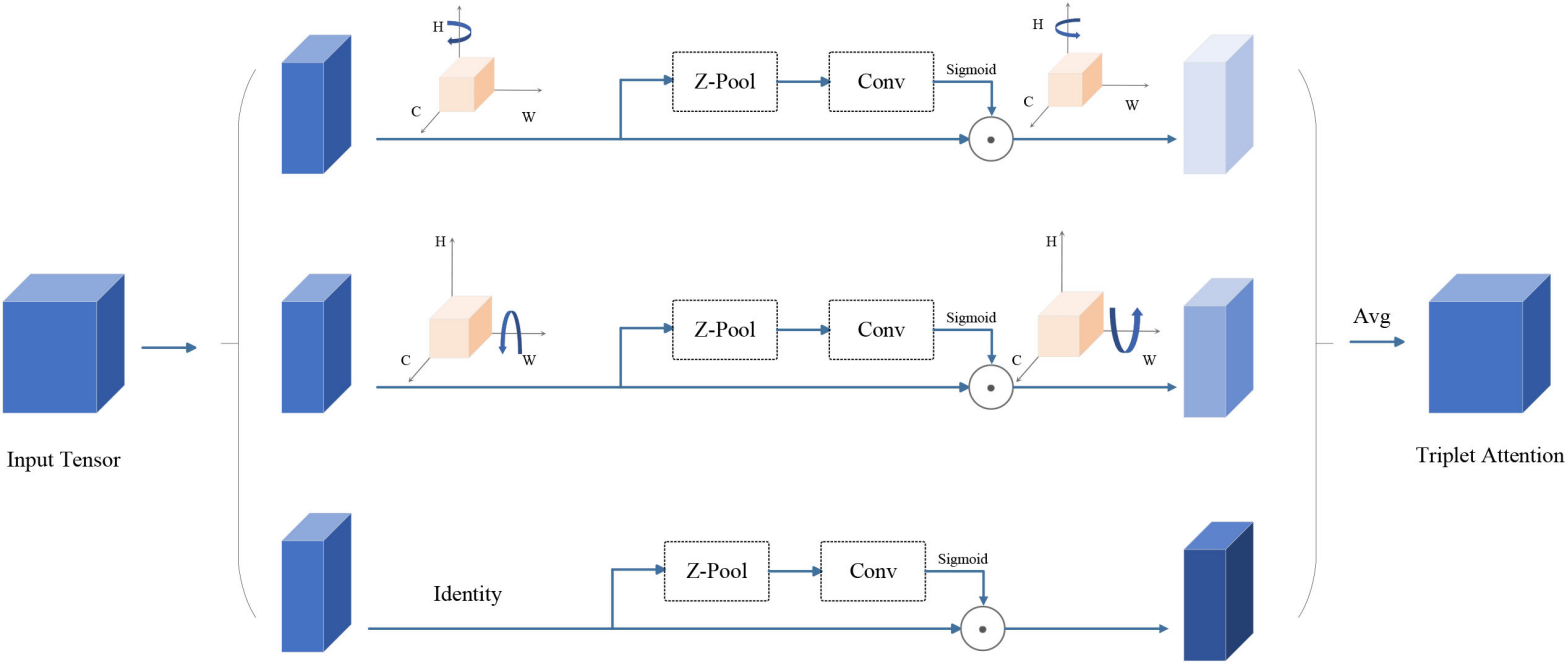
The Triplet Attention module.

As shown in **Figure 6**, Triplet Attention has three parallel branches. The first two branches are designed to capture the cross-dimensional interactions between channel C and spatial dimensions H and W. In the third branch, the input features are first processed through Zpooling, which is followed by the convolution layer, and finally, spatial attention weights are computed using the Sigmoid activation function. The output of these three branches is summed up to get the final attention map. Triplet Attention could reduce the information loss by modeling the interaction in different dimensions (channel height, channel width, and spatial dimensions) and then aggregation (Park et al., 2023). It can improve feature representation by mining specific parts while reducing the computational cost and error as little as possible without compromising too much.

The feature processing flow of the Star-Triplet Attention block is as follows. First, the input feature *F* is processed by a depthwise separable convolution to obtain the intermediate feature *x*. Then, *x* is transformed through two different branches. In the first branch, a convolution operation with ReLU activation and then batch normalization are applied to obtain *f_R_* (*x*). In the second branch, convolution and batch normalization are directly employed to obtain *f_C_* (*x*). Subsequently, the outputs of these two branches are then combined through element-wise multiplication to generate the feature representation *z*, which is then input into the Triplet Attention mechanism to enhance the feature expression capabilities across channels and spatial dimensions to generate *z*′. Then, *z*′ is processed by convolution and batch normalization to compute the new feature *v*. Finally, *v* is processed by a depthwise separable convolution, and element-wise addition is added to the original input feature *F* to form the final output *y*. The entire process integrates the depthwise separable convolution, the Triplet Attention mechanism, and residual connections to effectively enhance feature extraction capability and overall model performance. The calculation formulas are provided in **Equations 1**–**7**.


(1) $$x=f_{DWC}\left(F\right)$$

(2) $$f_{R}\left(x\right)=\text{BN}\left(\text{ReLU}\left(\text{Conv}\left(x\right)\right)\right)$$

(3) $$f_{C}\left(x\right)=\text{BN}\left(\text{Conv}\left(x\right)\right)$$

(4) $$z=f_{R}\left(x\right)\cdot f_{C}\left(x\right)$$

(5) $$z^{\prime }=TA\left(z\right)$$

(6) $$v=f_{C}\left(z^{\prime }\right)$$

(7) $$y=F+f_{DWC}\left(v\right)$$

Here, *F* represents the input feature, which is the output of the initial feature processing. The operation *f_DWC_* (·) denotes the depthwise separable convolution. The term *f_C_* (*x*) represents the operation of convolution followed by batch normalization, and *f_R_* (*x*) indicates the convolution operation with a ReLU activation function, followed by batch normalization. *z* represents the elementwise multiplication of two branches, and *TA*(·) signifies the triplet attention mechanism. *y* indicates the final output that incorporates a residual connection.

**Figure 7:**
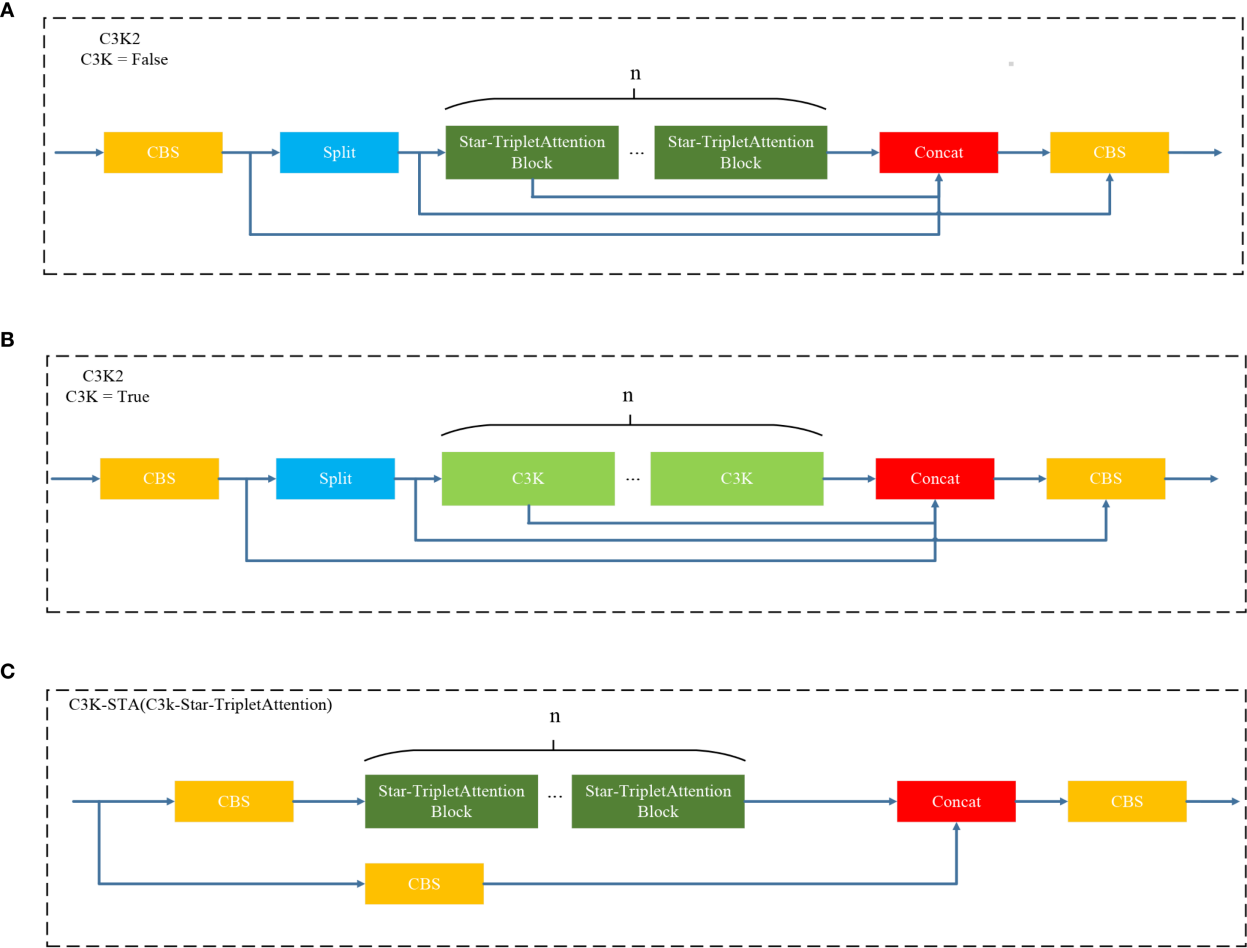
C3K2-STA module.

**Figure 7** illustrates the architecture diagram of the C3K2-STA module. This module utilizes Star Blocks for star operations and discards the original bottleneck structure. As such, it reduces redundant computations and the model size. Moreover, Star Blocks can obtain high-dimensional feature spaces from low-dimensional space inputs, which significantly enhances the ability to extract leaf disease features. By integrating the Triplet Attention mechanism and modeling multi-dimensional interactions (i.e., channels, spaces, and positions in parallel), this module simultaneously improves recognition performance while maintaining lightweight computation.

### 3.4 ADown

**Figure 8:**
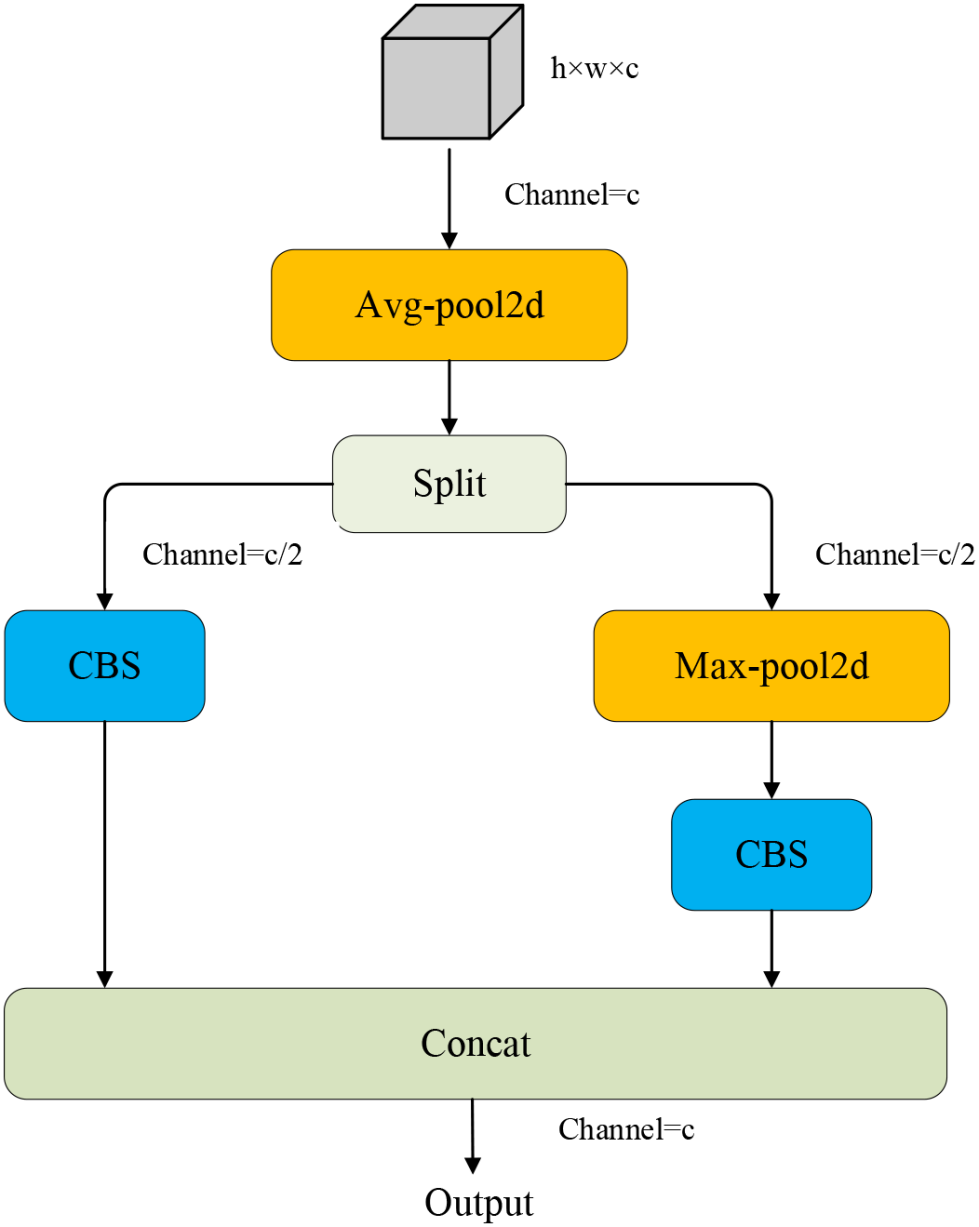
ADown module.

The ADown module (**Figure 8**) is a significant innovation in YOLOv9, introducing an efficient downsampling mechanism that enhances network depth and complexity without substantially increasing the number of parameters. This module combines average pooling and max pooling operations, which capture the global information, and the latter highlights local features (Zhang et al., 2024). Specifically, the input feature map undergoes average pooling and is then divided into two parts along the channel dimension. One part is directly convolved, while the other undergoes max pooling followed by convolution. At last, two feature maps are concatenated as the final output. Since the ADown module can extract both background information and edge information at the same time, it is applicable to leaf disease detection. In contrast, the network structure of YOLOv11 mainly relies on the Convolution-BatchNormScale (CBS) module for the downsampling. Although it can also realize effective feature extraction and non-linear transformation, many parameters bring more computational cost. Despite the kernel size, stride, and padding of this module being reasonably set, it still affects the inference cost. To alleviate the above problem, the CBS module in the backbone and neck of the model is replaced with the ADown module, which reduces computational cost and improves performance.

### 3.5 Wise-Inner-MPDIoU

In object detection, the core objective of bounding box regression is to optimize the predicted boxes so as to closely align with the ground truth (GT) annotations (He et al., 2019). The IoU has emerged as a widely adopted metric for evaluating the accuracy of these predictions (Rezatofighi et al., 2019). This metric assesses the degree of matching between the predicted box and the true box by computing the ratio of their intersection area to their union area. The mathematical formulation is given in Equation 8.


(8) $$IoU=\frac{\left|B_{p}\cap B_{g}\right|}{\left|B_{p}\cup B_{g}\right|}$$

where *B_p_* and *B_g_* represent the area of the predicted bounding box and the ground truth bounding box, respectively. However, YOLOv11 adopts the CIoU loss for regression due to its obvious limitations when the loss presents multi-factor improvements on the performance of the bounding box (Feng and Jin, 2024). Specifically, when the width-to-height ratio of the predicted box is linearly proportional to that of the ground truth box, the width-to-height ratio penalty (expressed as a relative value) in CIoU becomes ineffective, resulting in slower convergence. In addition, the inverse trigonometric function used in CIoU leads to high computational cost during training, which may degrade the overall efficiency.

In response to these limitations, this study attempts to alleviate these issues by designing the Wise-Inner-MPDIoU loss function. Differing from previous loss functions, the Wise-Inner-MPDIoU loss function combines a dynamic weighting strategy of WIoU, geometric precision of MPDIoU, and inner region sensitivity of Inner-IoU in a synergistic way. WIoU adopts a non-monotonic focusing way and adaptive gradient allocation to alleviate the harmful gradient from outliers and dynamically weigh overlapping areas to reduce the deviation of position (Du et al., 2023). The Inner-IoU component enhances localization accuracy by prioritizing internal overlap quality through a minimum-area normalization strategy. It replaces the union area used in IoU in previous methods with the smaller area of the two bounding boxes, which enhances the sensitivity to small targets or occluded targets. MPDIoU further improves the method by enforcing exact corner-point alignment between predicted and ground truth boxes, which solves the convergence delay problem brought by the aspect ratio dependencies of CIoU (Cao et al., 2024).

WIoU v3 is chosen as the preferred variant in this study to extend the distance-attention framework of WIoU v1 with a non-monotonic focusing coefficient (*γ*) and lower gradient gains due to the low quality of the samples. By computing a distance-based weight *R_WIoU_* to modulate the IoU loss, the formulation of WIoU is defined in **Equations 9**–**11**.


(9) $$L_{IoU}=1-IoU$$

(10) $$R_{WIoU}=\text{exp }\left(\frac{{\left(x-x_{gt}\right)}^{2}+{\left(y-y_{gt}\right)}^{2}}{W_{g}^{2}+H_{g}^{2}}\right)$$

(11) $$L_{WIoU_{v1}}=R_{WIoU}*L_{IoU}$$

For the predicted bounding box, *x* and *y* denote the predicted values of the center coordinates, while *x*_gt_ and *y*_gt_ represent the center coordinates of the true bounding box. Furthermore, *W_g_* and *H_g_* indicate the widths and heights, respectively, of the minimum enclosing rectangle in the anchor box and the target box. By designing *L*_IoU_, the anchor box of poor quality can be enhanced. When *R*_WIoU_ is used in distance measurement, it can suppress the attention of anchor boxes of high quality and alleviate the over-dependence on centroid distance (Xiong et al., 2024). The formula definition of WIoU v3 is given in **Equations 12**–**14**.


(12) $$\beta =\frac{L_{IoU}^{*}}{L_{IoU}}\in \left[0,+\infty \right)$$

(13) $$\gamma =\frac{\beta }{\delta \alpha \beta -\delta }$$

(14) $$L_{WIoU_{v3}}=\gamma L_{WIoU_{v1}}$$

where *β* is the outlier value that represents the anchor box’s description degree of goodness. In other words, the larger outlier value *β* represents the worse quality of anchor boxes. The hyperparameters *α* and *δ*, together with outlier degree *β*, are used to determine the non-monotonic focusing coefficient *γ*. The coefficient *γ* can decrease the competitiveness of good samples and, at the same time, weaken the harmful gradients caused by poor samples. Therefore, WIoU v3 can non-monotonically and dynamically focus on the ordinary samples and improve the generalization ability and the overall performance of the model.

Inner-IoU is designed to enhance localization accuracy by optimizing the overlapping area of the predicted box and the ground truth box (Ding et al., 2019). Since the minimum area is used as the normalization denominator, the loss function is more sensitive to the alignment of the target’s internal structure. The formula is shown in Equation 15.


(15) $$L_{\text{Inner}-\text{IoU}}=1-\frac{\left|B_{p}\cap B_{g}\right|}{min\;(\left|B_{p}\left|,\right|B_{g}\right|)}$$

where *B_p_* and *B_g_* are the areas of the predicted bounding box and the ground truth bounding box, respectively. |*B_p_* ∩ *B_g_* | is the intersection area between *B_p_* and *B_g_*, and min(|*B_p_* |,|*B_g_* |) is the smaller one between |*B_p_* | and |*B_g_* |. By substituting the union area (conventional in traditional IoU) with the minimum area as the denominator, Inner-IoU significantly enhances sensitivity to small objects and occluded scenarios. Even a slight shift in the position of the predicted box will result in a large change in the minimum-area denominator, which encourages more accurate bounding box localization.

MPDIoU incorporates the distance between matching corner points of the predicted and true bounding boxes. As a result, it facilitates more precise geometric alignment during training. This module has been shown to enhance the accuracy of regionalization by integrating the corner distance concept into the intersection over union. The mathematical formulation is expressed in Equation 16.


(16) $$L_{MPDIoU}=1-\text{IoU}+\frac{d_{1}^{2}}{w^{2}+h^{2}}+\frac{d_{2}^{2}}{w^{2}+h^{2}}$$

where *d*_1_ and *d*_2_ indicate the distances between the top-left and bottom-right corner coordinates of the ground truth bounding box and the predicted bounding box, respectively. *w* and *h* represent the width and height, respectively, of the final output feature map of the network. By taking into account the positional discrepancies of the corner points between the predicted and ground truth bounding boxes, MPDIoU guides the model to adjust the positions of the bounding boxes with greater accuracy, thus improving the stability of target localization.

The formula for Wise-Inner-MPDIoU is expressed in **Equation 17**.


(17) $$L_{\text{Wise}-\text{Inner}-\text{MPDIoU}}=\left(1-{\text{WIoU}}_{\text{v}3}\right)+\lambda_{1}\cdot \frac{d_{1}^{2}+d_{2}^{2}}{w^{2}+h^{2}}+\lambda_{2}\cdot \left(1-\frac{\left|B_{p}\cap B_{g}\right|}{min\;(\left|B_{p}\left|,\right|B_{g}\right|)}\right)$$

Here, *λ*_1_ and *λ*_2_ are used to adjust the weight contributions of different constraint terms. This fusion strategy enables Wise-Inner-MPDIoU to jointly evaluate the spatial alignment and positional discrepancies between predicted and ground truth bounding boxes more effectively. It mitigates the overemphasis on both high-quality anchor boxes (with low localization loss) and low-quality anchor boxes (with high localization loss), thereby facilitating the optimization of moderately performing anchor boxes. This approach ensures convergence speed while effectively boosting the precision of object bounding box regression.

## 4 Analysis and interpretation of experimental results

### 4.1 Experimental platform

The experiments are carried out on a server with the following hardware and software configurations: two RTX 4090 GPUs with a total of 48 GB GPU memory and an Intel 8360Y CPU. The platform is equipped with Ubuntu 22.04 LTS, CUDA 11.8, Python 3.9.19, PyTorch 2.0.1, and PyCharm 2020.3. The specific configuration is shown in **Table 1**.

**Table 1:** Hardware and software configuration used in the experiment.

Software and hardware	Version or model
Operating system	Ubuntu 22.04 LTS
CPU	Intel 8360Y
GPU	2 × NVIDIA GeForce RTX 4090
Display memory	48G
CUDA	11.8
PyTorch version	2.0.1
Python version	3.9.19
Software	PyCharm 2020.3

During the training phase, the YOLO-Citrus model adopts transfer learning to accelerate the training speed. Specifically, the number of training epochs is set to 200 with a batch size of 16, and the Stochastic Gradient Descent (SGD) optimizer is employed. The remaining parameters are set to the default configuration of the official YOLOv11s model. Moreover, an early stopping strategy is implemented, which terminates the training process if the model’s performance fails to improve after 100 epochs.

### 4.2 The assessment metrics for the network model

This study evaluates the performance of citrus disease detection using the following metrics: model size, Giga Floating Point Operations per Second (GFlops), precision, recall, F1 score, average precision (AP), and mean average precision (mAP). These parameters are widely used in object detection methods to characterize the citrus disease detection accuracy. The definitions of these parameters are shown in **Equations 18**–**22**.


(18) $$\text{Precision }=\;\frac{TP}{TP\;+\;FP}$$

(19) $$\text{Recall}\;=\;\frac{TP}{TP\;+\;FN}$$

(20) $$F1=2\;\times \frac{\text{Precision }\cdot \text{ Recall}}{\text{Precision }+\text{ Recall}}$$

(21) $$AP\;=\int_{0}^{1}\left(\text{Precision}\cdot \text{Recall}\right)\;d\text{Recall}$$

(22) $$mAP=\frac{1}{N}\sum_{i=1}^{N}AP_{i}$$

where TP (True Positive) is the number of diseased leaves that are detected by the model. FP (False Positive) occurs when a healthy leaf or a leaf with a different disease is incorrectly classified as having the target disease. FN (False Negative) represents the diseased leaves that the model fails to identify. Higher Precision implies more accurate judgments made by the model. Recall is the ratio of actual positive samples that are identified by the model. The F1 score is a metric that integrates precision and recall into a weighted average. AP gives an overall idea of the model’s performance by calculating precision for all the values of recall. mAP represents the average of AP over all the diseases, an overall measurement of the model’s capability of detecting different diseases. Thus, all these metrics give an overall idea of the model’s performance.

### 4.3 Performance analysis of the benchmark model

For the performance analysis of various YOLOv11 models for citrus leaf disease, a test is performed for each YOLOv11 on the constructed dataset. The test results are shown in **Table 2**. From the test results, it can be found that there are obvious differences in terms of model size and computational efficiency among the YOLOv11 variants. If balance is made between model size, computational efficiency, and detection accuracy, YOLOv11s gets the best performance. Its model size is 19.2 MB, computational load is 21.3 GFlops, and mAP@0.5 is 95.2%. YOLOv11n has the smallest model size of 5.5 MB and the smallest computational load of 6.3 GFlops, and its mAP@0.5 is 93.0%, which is significantly lower than that of YOLOv11s. YOLOv11m achieves the highest mAP@0.5 with 96.5%, but its model size is 40.5 MB and the computational load is 67.7 GFlops, approximately 2.1 times and 3.2 times, respectively, higher than those of YOLOv11s. This leads to a substantial increase in resource requirements. The mAP@0.5 values of YOLOv11l and YOLOv11x are 96.1% and 95.9%, respectively. However, their model sizes are 51.2 MB and 114.4 MB, and their computational loads are 86.6 and 194.4 GFlops, respectively. These are approximately 2.7 and 6.0 times, and 4.1 and 9.1 times greater than those of YOLOv11s, respectively, indicating lower efficiency. Therefore, based on the balanced consideration of model efficiency and detection performance, this study selects YOLOv11s as the benchmark model for object detection tasks. It can still maintain a high detection accuracy, while its model size and computational requirements are smaller. As such, it is suitable for efficient deployment in practical applications.

**Table 2:** Performance comparison of YOLOv11 models.

Model	mAP@0.5 (%)	Size (MB)	GFlops
YOLOv11n	93.0	5.5	6.3
YOLOv11s	95.2	19.2	21.3
YOLOv11m	96.5	40.5	67.7
YOLOv11l	96.1	51.2	86.6
YOLOv11x	95.9	114.4	194.4

### 4.4 Analysis of the ablation experimental results

**Figure 9:**
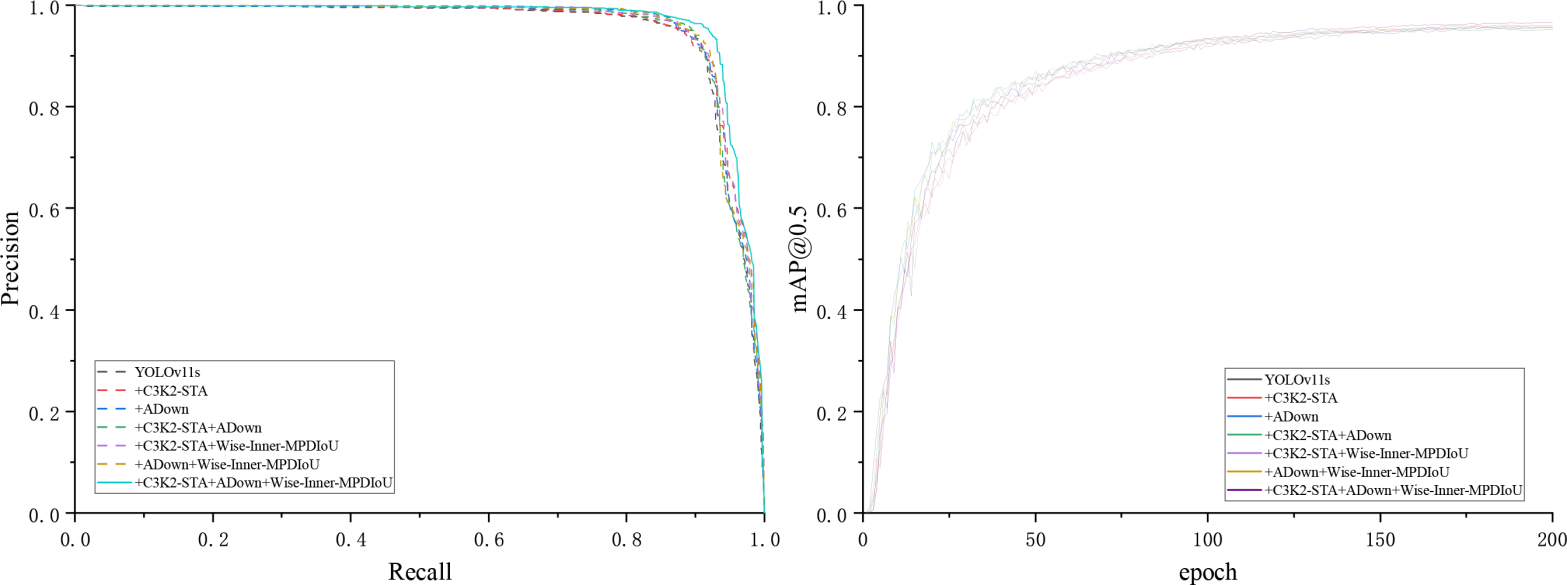
P–R curves and mAP@0.5 results of the improved YOLOv11s model under different configurations.

As shown in **Table 3**, all these parts are beneficial to the final performance of YOLOv11s. The baseline model (using only YOLOv11s) attains a mAP@0.5 of 95.2% and a mAP@0.5:0.95 of 80.2%, with a model size of 19.2 MB and a computing cost of 21.3 GFlops. When the C3K2-STA module is added individually, mAP@0.5 is enhanced to 95.7%, while mAP@0.5:0.95 is improved to 81.1%. Model size and computation cost are decreased slightly. It means that the C3K2-STA module can boost both detection accuracy and model efficiency at the same time. The ADown module can decrease model complexity dramatically while maintaining detection accuracy. Following the incorporation of the ADown module, mAP@0.5 persists at 95.7%, while mAP@0.5:0.95 ascends to 81%, accompanied by a substantial decline in model size and computation to 15.4 MB and 17.2 GFlops, respectively. When C3K2-STA and ADown are applied simultaneously, mAP@0.5 is decreased slightly to 95.6%, while mAP@0.5:0.95 is further improved to 81.8%. Model size and computation are decreased to 14.4 MB and 17.0 GFlops. It means that C3K2-STA and ADown have a synergistic effect on improving detection accuracy and decreasing model complexity. By combining the Wise-Inner-MPDIoU loss function with the C3K2-STA and ADown modules, mAP@0.5 improves in both cases, enhancing the model’s performance at lower IoU thresholds. In summary, the individual or combined use of the C3K2-STA, ADown, and Wise-Inner-MPDIoU modules can effectively boost the detection accuracy and model efficiency of YOLOv11s. In particular, when integrating the C3K2-STA, ADown, and Wise-Inner-MPDIoU modules together, mAP@0.5 is improved to 96.6%, while mAP@0.5:0.95 is improved to 81.6%. Model size and computation are decreased to 14.4 MB and 17.0 GFlops, respectively. This approach has been shown to significantly reduce model complexity and computational requirements while maintaining high levels of performance. Consequently, these modules are instrumental in enhancing the citrus disease recognition model proposed in this study. **Figure 9** compares the P–R curves and mAP@0.5 results of the enhanced YOLOv11s model under different configurations, highlighting the impact of our proposed modifications.

**Table 3:** Ablation experimental results.

YOLOv11s	C3K2-STA	ADown	Wise-Inner-MPDIoU	mAP@0.5 (%)	mAP@0.5:0.95 (%)	Size (MB)	GFlops
✓	×	×	×	95.2	80.2	19.2	21.3
✓	✓	×	×	95.7	81.1	18.2	21.1
✓	×	✓	×	95.7	81.0	15.4	17.2
✓	✓	✓	×	95.6	81.8	14.4	17.0
✓	✓	×	✓	96.0	80.8	18.2	21.1
✓	×	✓	✓	95.9	80.7	15.4	17.2
✓	✓	✓	✓	96.6	81.6	14.4	17.0

### 4.5 Performance evaluation of the YOLO-Citrus model

**Figure 10:**
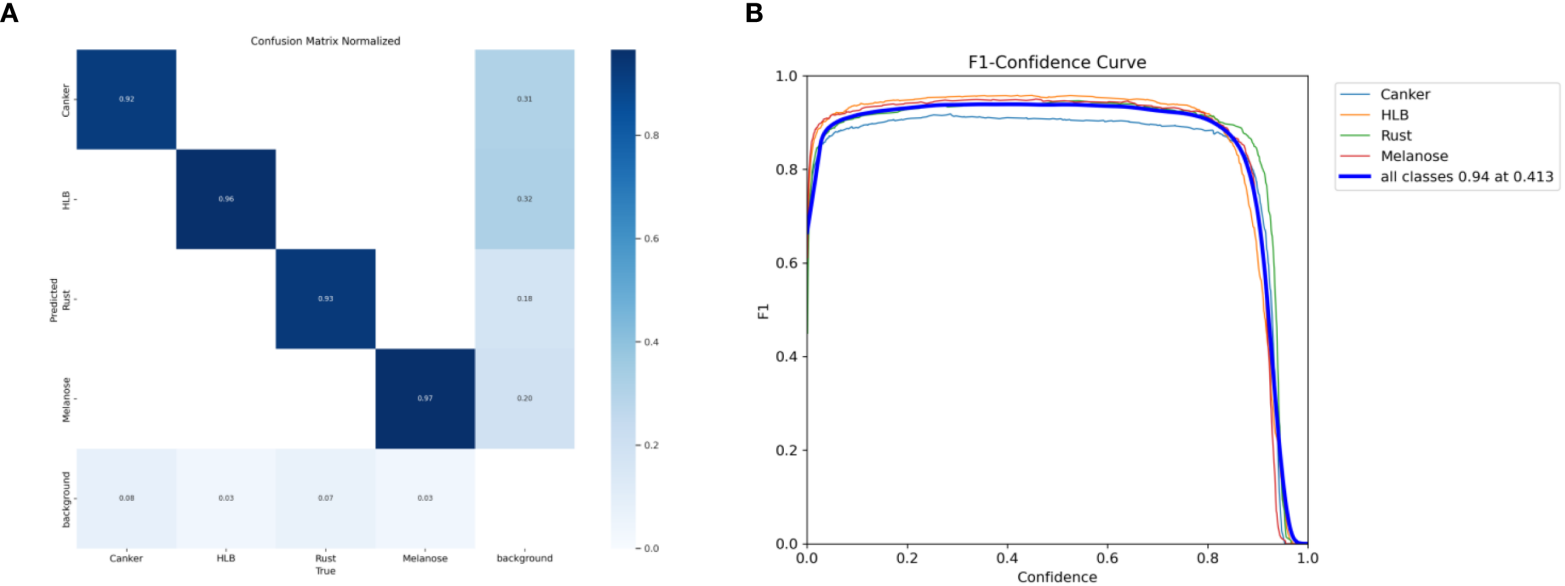
**(A)** Normalized confusion matrix and **(B)** F1-confidence curve of the YOLO-Citrus model under different configurations.

To further analyze the overall effect of YOLO-Citrus in multi-class citrus disease recognition, a normalized confusion matrix is constructed based on the test set. Additionally, the F1 score curve is utilized to further assess the overall performance of the model. The corresponding visualization results are presented in **Figure 10**, which illustrates the classification accuracy across four major disease categories as well as the variation of F1 score under different confidence thresholds.

As shown in **Figure 10**, the normalized diagonal accuracies of the four main disease categories are 0.92, 0.96, 0.93, and 0.97. This indicates that there is little misclassification between categories and that YOLO-Citrus has stable recognition performance. When the confidence threshold is 0.413, the weighted average F1 score is 0.94. This also indicates that YOLO-Citrus can achieve a balanced overall precision and recall.

From the above analysis, it can be seen that there is still a low misclassification rate in most categories and that YOLO-Citrus performs well in the category of concern, which indicates that YOLO-Citrus has strong practical application potential. Although there are still some false positives and false negatives in most categories, YOLO-Citrus has a high overall detection performance and can meet the practical application requirements of intelligent diagnostic systems. In some cases, there may be misclassifications between categories. These misclassifications are caused by the fact that the visual appearance of different disease symptoms is not significantly different. This type of confusion is biologically plausible and may affect the timeliness of early diagnosis and treatment in real-world applications.

Therefore, YOLO-Citrus has excellent overall performance, strong class-wise recognition ability, and strong reliability, which provide effective technical support for the early detection and intelligent management of citrus diseases.

### 4.6 The comparative analysis mainstream object detection models

**Figure 11:**
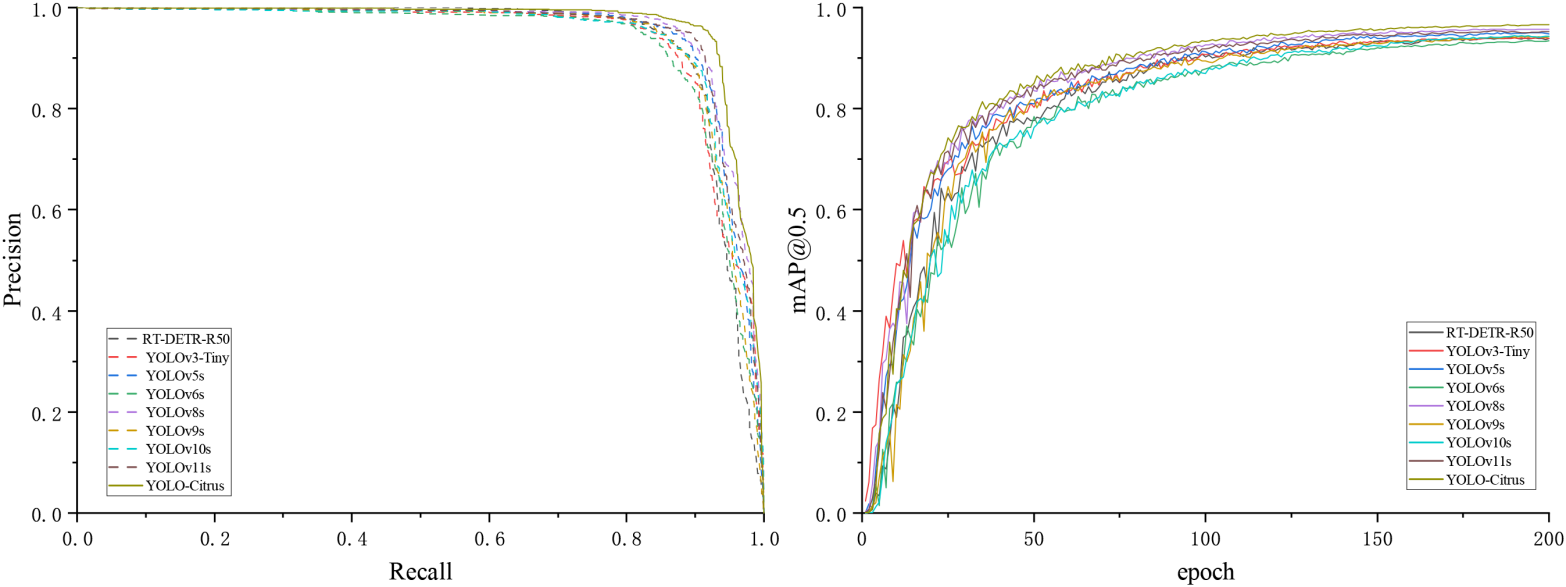
Detection results of the P–R curve and mAP@0.5 curve of mainstream object detection algorithms.

This subsection presents a comparative analysis of several mainstream object detection models, including RT-DETR-R50, YOLOv3-Tiny, YOLOv5s, YOLOv6s, YOLOv8s, YOLOv9s, YOLOv10s, YOLOv11s, and YOLO-Citrus. The specific outcomes are collectively illustrated in **Table 4**, as well as in **Figure 11**.

**Table 4:** Comparative experimental results.

Model	Precision (%)	Recall (%)	mAP@0.5 (%)	mAP@0.5:0.95 (%)	Size (MB)	Parameters	GFlops
RT-DETR-R50	93.1	89	93.9	79.5	82.0	41,942,904	125.6
YOLOv3-Tiny	93.1	89.7	94.0	74.8	19.2	9,512,080	14.3
YOLOv5s	93.6	89.2	95.0	77.6	15.9	7,815,164	18.7
YOLOv6s	93.9	84.8	93.3	74.0	32.2	15,976,924	42.8
YOLOv8s	94.5	91.5	95.7	80.8	19.9	9,829,212	23.4
YOLOv9s	94.1	87.3	94.2	77.4	13.3	6,195,196	22.1
YOLOv10s	92.2	88.5	94.4	75.2	16.5	7,219,548	21.4
YOLOv11s	95.0	91.1	95.2	80.3	19.2	9,414,348	21.3
YOLO-Citrus	95.1	93.0	96.6	81.6	14.4	6,947,156	17.0

**Table 4** shows the precision, recall, mAP@0.5, mAP@0.5:0.95, model size, number of parameters, and computational load of these models. The results indicate that YOLO-Citrus outperforms the other models across multiple evaluation metrics. YOLO-Citrus gets mAP@0.5 of 96.6% and mAP@0.5:0.95 of 81.6%; meanwhile, the model size is only 14.4 MB, and the computational load is only 17.0 GFlops. It shows that YOLO-Citrus has good lightweight characteristics in addition to high accuracy, which is suitable for deployment on a device with limited resources.

RT-DETR-R50 gets a mAP@0.5 of 93.9% and a mAP@0.5:0.95 of 79.5%. However, it needs 41.94M parameters and 125.6 GFlops, which are much more than those of YOLO-Citrus. Despite this higher resource consumption, its detection accuracy is still lower. YOLOv3-Tiny achieves a mAP@0.5 of 94.0%, and YOLOv10s reaches 94.4%, both of which are respectively 2.6% and 2.2% lower than those of YOLO-Citrus’s 96.6%. Their recall rates are respectively 89.7% and 88.5%, which are 3.3% and 4.5% lower than those of YOLO-Citrus’s 93.0%. YOLOv6s has a mAP@0.5 of 93.3%, 3.3% lower than that of YOLO-Citrus, but its computational load of 42.8 GFlops and parameter count of 15.98M are 2.52 times and 2.3 times those of YOLO-Citrus, respectively. YOLOv8s obtains a mAP@0.5 of 95.7%, which is a 0.7% improvement compared with that of YOLOv5s. In addition, YOLOv8s gets a mAP@0.5:0.95 of 80.8%, which is a 3.2% improvement compared with that of YOLOv5s. However, its model size is 19.9 MB and its computation load is 23.4 GFlops, which are respectively 38.2% and 37.6% larger than those of YOLO-Citrus. YOLO-Citrus achieves a mAP@0.5 of 96.6% with 6.95M parameters, reducing the parameter count of the original YOLOv11s by 26.2% (from 9.41M) and improving the accuracy by 1.4%. Those results reach the best balance in terms of detection accuracy, amount, and computation load.

**Figure 12:**
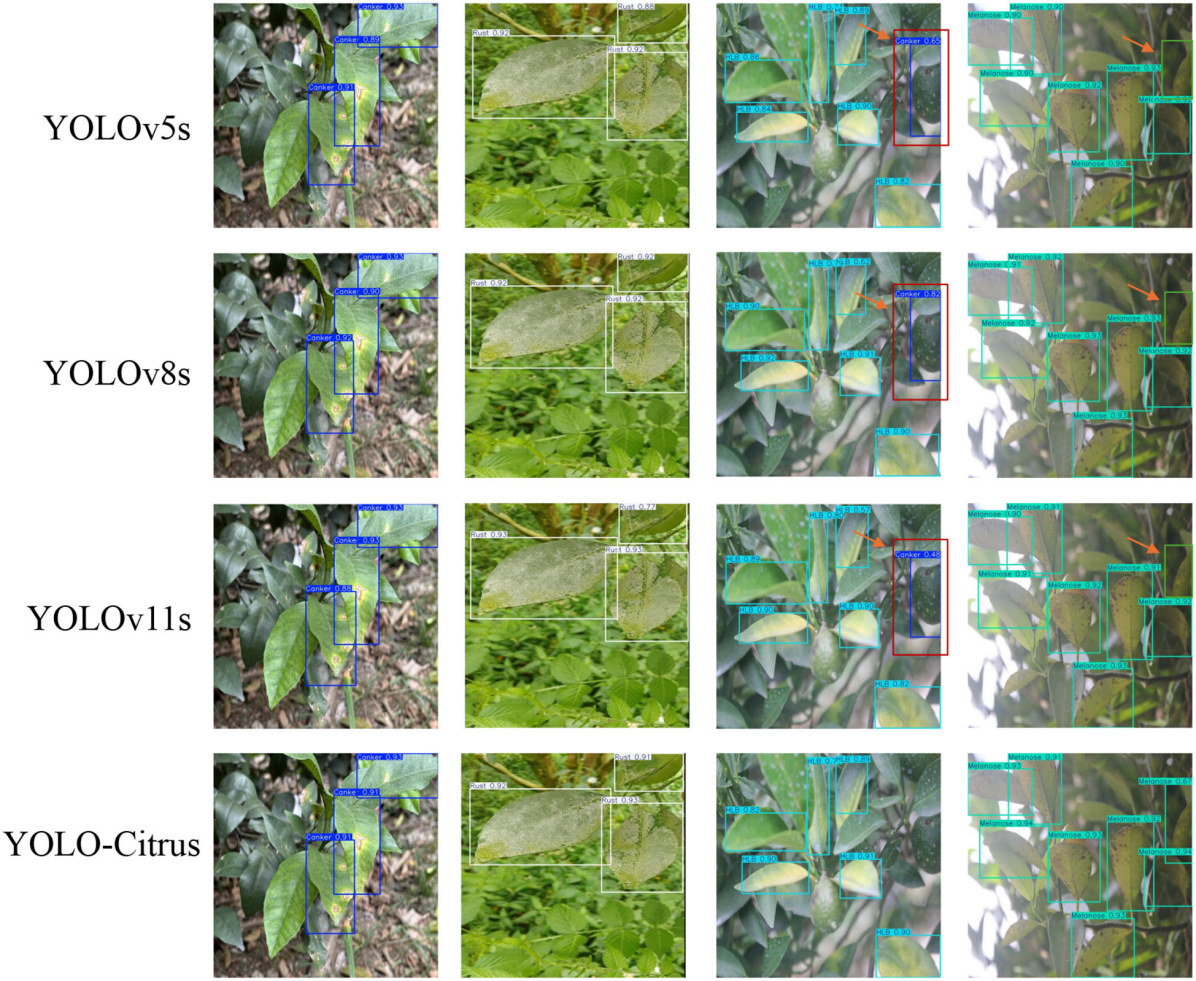
Test results of different models on test images. Red boxes indicate false detections, and green boxes indicate missed detections.

As shown in **Figure 11**, the P–R curve of YOLO-Citrus always converges to the upper-right corner closer than other models, which means that YOLO-Citrus is better than other models in terms of the precision–recall curve. Furthermore, YOLO-Citrus obtains the highest mAP@0.5 among all models, which is also evidence that YOLO-Citrus is the best. In addition, as displayed in **Figure 12**, the detection results of other models on some test images are displayed, where the red boxes represent the false detections and green boxes represent the missed detections. These test images are derived from our dataset and encompass several prevalent citrus leaf diseases, including canker, rust, Huanglongbing, and melanose. YOLO-Citrus has fewer localization errors and missed targets than other models, which shows that YOLO-Citrus is more robust in practical applications.

### 4.7 Model deployment experiments

**Figure 13:**
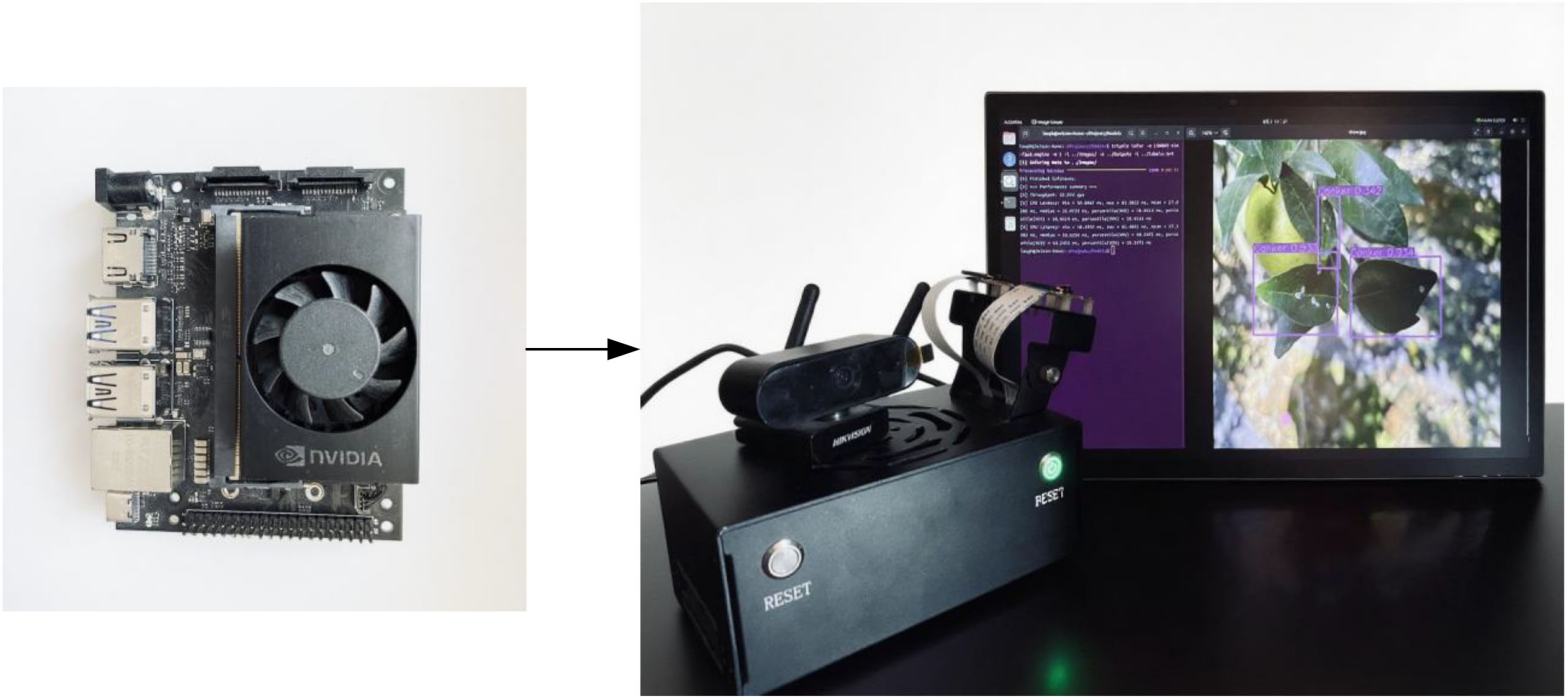
Edge platform configuration and testing outcomes.

Finally, YOLO-Citrus is run on an NVIDIA Jetson Orin Nano 8 GB edge device, with 40 TOPS computing power. The device runs on the JetPack 6.1 software platform and uses TensorRT technology for acceleration, with the model optimized using FP16 precision to enhance inference efficiency. During the deployment, image data are collected using a Hikvision DS-E11 camera, and the detection results are visualized in real time on the 15-inch touch screen to construct an integrated visual inspection system, as shown in **Figure 13**. As shown in the experimental results, the inference speed of the model can remain at a relatively stable level of 51.5 FPS, and the standard deviation is very small. Most of the images are finished within 18.4 ms, which shows the real-time performance of the model. These results show that YOLO-Citrus can run in a resource-constrained edge environment efficiently and stably. The real-time disease detection system has been constructed and implemented on citrus orchards, providing evidence to support timely decision-making in intelligent agriculture.

## 5 Conclusions

This paper presents the YOLO-Citrus network model designed for intelligent detection of citrus leaf diseases. Through the realization of automatic disease identification and early warning, a large part of the citrus cultivation management process, the intelligence of the next step is significantly promoted, and the yield and quality of citrus fruits are improved at the same time.

Specifically, three novel technical components are introduced into the YOLO-Citrus model. First, the dynamically enhanced C3K2-STA module merges Star Block’s dynamic receptive fields with Triplet Attention’s cross-dimensional attention mechanism. Second, the computationally efficient ADown downsampling strategy integrates dual-path pooling with axial feature reorganization. Third, the geometrically optimized Wise-Inner-MPDIoU loss function implements dynamic weight allocation with inner-product distance metrics.

The experimental results verify the superiority of the model. YOLO-Citrus achieves a mAP@0.5 of 96.6%, which is an improvement of 1.4 percentage points compared with the baseline YOLOv11s. Additionally, it attains a mAP@0.5:0.95 of 81.6%, surpassing the YOLOv11s benchmark of 80.3%. Detailed comparison with other state-of-the-art models, such as YOLOv5s, YOLOv8s, and YOLOv11s, on evaluation metrics, shows that YOLO-Citrus outperforms other models in terms of detection performance.

Remarkably, without sacrificing outstanding detection accuracy, YOLO-Citrus obtains significant improvements on both computational efficiency and model compactness. The architecture achieves a 26.2% reduction in parameters (6.9M), a 20.2% decrease in computational cost (17.0 GFlops), and compresses the model size by 25.0% to 14.4 MB, compared to the 19.2 MB baseline. These optimizations provide significant advantages for deployment in resource-constrained agricultural environments.

To verify its practical usability, YOLO-Citrus is deployed on a Jetson Orin Nano edge device. The maximum FPS is 51.5 with low latency (18.4 ms per image) and steady state, which means that YOLO-Citrus can be used for real-time on-site detection without depending on high infrastructure. Thus, farmers can obtain feedback in the field immediately and take corresponding actions in time to prevent the disease from spreading further, which helps reduce pesticide misuse, lower costs, and improve yield through more informed decision-making.

In summary, YOLO-Citrus establishes new performance standards for disease detection models while delivering practical solutions for intelligent citrus disease management. This research not only contributes a high-precision, efficient lightweight detection framework covering multiple major citrus diseases, including canker, rust, melanose, and HLB, but also demonstrates its real-world agricultural applications. Considering the severe economic and agronomic impacts of diseases such as HLB, real-time, leaf-level detection frameworks like YOLO-Citrus offer promising tools for early diagnosis and effective management in citrus orchards. Future investigations will focus on enhancing environmental adaptability and extending the model’s capabilities to other crop disease detection scenarios, thereby broadening the technology’s impact and applicability.

## Data Availability

The raw data supporting the conclusions of this article will be made available by the authors, without undue reservation.
